# Chlorpromazine Protects Against Apoptosis Induced by Exogenous Stimuli in the Developing Rat Brain

**DOI:** 10.1371/journal.pone.0021966

**Published:** 2011-07-14

**Authors:** Jing Wu, Rongrong Song, Wuqi Song, Yujun Li, Qingmeng Zhang, Yang Chen, Yingmei Fu, Wenjuan Fang, Jindong Wang, Zhaohua Zhong, Hong Ling, Liming Zhang, Fengmin Zhang

**Affiliations:** 1 The Heilongjiang Key Laboratory of Immunity and Infection, Pathogenic Biology, Department of Microbiology, Harbin Medical University, Harbin, Heilongjiang, China; 2 Department of Neurology, The Affiliated Hospital, Harbin Medical University, Harbin, Heilongjiang, China; 3 Key Laboratory of Bio-Pharmaceutical, Harbin Medical University, Ministry of Education, Harbin, Heilongjiang, China; University of Queensland, Australia

## Abstract

**Background:**

Chlorpromazine (CPZ), a commonly used antipsychotic drug, was found to play a neuroprotective role in various models of toxicity. However, whether CPZ has the potential to affect brain apoptosis *in vivo* is still unknown. The purpose of this study was to investigate the potential effect of CPZ on the apoptosis induced by exogenous stimuli.

**Methodology:**

The ethanol treated infant rat was utilized as a valid apoptotic model, which is commonly used and could trigger robust apoptosis in brain tissue. Prior to the induction of apoptosis by subcutaneous injection of ethanol, 7-day-old rats were treated with CPZ at several doses (5 mg/kg, 10 mg/kg and 20 mg/kg) by intraperitoneal injection. Apoptotic cells in the brain were measured using TUNEL analysis, and the levels of cleaved caspase-3, cytochrome c, the pro-apoptotic factor Bax and the anti-apoptotic factor Bcl-2 were assessed by immunostaining or western blot.

**Findings:**

Compared to the group injected with ethanol only, the brains of the CPZ-pretreated rats had fewer apoptotic cells, lower expression of cleaved caspase-3, cytochrome c and Bax, and higher expression of Bcl-2. These results demonstrate that CPZ could prevent apoptosis in the brain by regulating the mitochondrial pathway.

**Conclusions:**

CPZ exerts an inhibitory effect on apoptosis induced by ethanol in the rat brain, intimating that it may offer a means of protecting nerve cells from apoptosis induced by exogenous stimuli.

## Introduction

Chlorpromazine (CPZ), a phenothiazine neuroleptic drug, has a very wide range of applications in the treatment of psychosis, anti-emesis and the induction of artificial hibernation [1]. Over the years, the evidence regarding protective effects of CPZ on the nervous system is supported by the *in vitro* and animal studies reporting its beneficial effects in various models of toxicity, including ischemia [2], β-amyloid protein-induced Ca^2+^ uptake [3], cyanide poisoning [4] and glutamate-induced neurotoxicity [5]. Moreover, recent studies suggest that CPZ treatment leads to a higher level of the anti-apoptotic factor Bcl-2 level in the schizophrenic cortexes of treated subjects compared to antipsychotic-naive subjects [6].

These findings suggest that, apart from its antagonistic actions on dopamine receptors, CPZ may also play a role in the regulation of apoptosis during the course of drug treatment. In the present study, ethanol-treated seven-day-old rats were used as an apoptosis model to determine the potential effect of CPZ on ethanol-induced apoptosis in the brain by measuring the number of apoptotic cells and the expression of apoptosis-related proteins of the mitochondrial pathway.

## Materials and Methods

### Ethics Statement

All animal procedures performed in this work followed guidelines in accordance with the Regulations for the Administration of Affairs Concerning Experimental Animals, and approved by the animal care and welfare committee of Harbin Medical University (Protocol number of Animal Experimental Ethical Inspection 2009104).

### Animals and treatment

Wistar rats were obtained from the Center for Laboratory Animals, Harbin Medical University, China. All the animals were housed in the departmental animal colony in a vivarium with a controlled climate (temperature 22°C, 30% humidity) and a 12-h light/dark cycle.

Seven-day-old rats were injected intraperitoneally with CPZ (Shanghai Harvest Pharmaceutical CO., LTD., China) at various doses (5 mg/kg, 10 mg/kg and 20 mg/kg). 24 h after CPZ injection, rats were injected subcutaneously with 20% ethanol diluted in saline solution, to a final dose of 5.0 g/kg body weight, according to the method described by Ikonomidou [7]. The rats were treated with CPZ at the beginning of postnatal day seven, and were exposed to ethanol at the end of day seven. The rats in each treatment group are of the same age. The rats were euthanized by decapitation without anesthesia to avoid the contamination of brain with chemicals. The rats were sacrificed at several time points after ethanol exposure (4 h, 8 h, 12 h or 24 h). Saline injections of equal volume were used as controls. Animals in each litter were distributed into the following treatment groups: saline control, ethanol, CPZ pretreatment + ethanol. In addition, to determine the effect of CPZ on apoptosis-related protein expression in the brain, the rats were injected intraperitoneally with CPZ (10 mg/kg) once daily for two weeks or were given a single injection of a high dosage (20 mg/kg). A total of 150 infant rats were used for the entire study, and four to nine animals from different litters were used for each data point.

### TUNEL assay

Terminal deoxynucleotidyl transferase-mediated dUTP nick end-labeling (TUNEL) was performed at 24 h after ethanol treatment. Rat brain samples were fixed, dehydrated, embedded in paraffin, and sectioned into 4-µm slices prior to TUNEL and immunohistochemical staining. To detect apoptotic cells, the TUNEL reaction mixture was applied to the paraffin sections using an *in situ* cell death detection kit POD (Roche, Germany) as described by the manufacturer. After deparaffinization, the sections were treated with 20 µg/ml proteinase K for 10 min. After treatment with 0.3% H_2_O_2_ in methanol for 10 min, the sections were incubated with the TUNEL reaction mixture for 60 min at 37°C. Further incubation with peroxidase-conjugated antibody was performed for 30 min at 37°C. The sections were stained with diaminobenzidine (DAB) solution for 10 min at room temperature and then counterstained with hematoxylin. For negative controls, the TUNEL reaction mixture was omitted, and the same staining procedures were followed.

The sections were then visualized using a microscope (CX31, Olympus, Japan), and digital photographs were taken with a camera (MicroPublisherTM 5.0 RTV, Olympus, Japan). For each group, all of the apoptotic cells throughout the whole cortex and the hippocampus were counted under the microscope. Follow the example of microscope counting chamber; we marked the slides with grid lines to separate the section into many small squares. Total numbers of the TUNEL-positive cells were counted under the microscope with a hand-held counter. The cells touching the middle line were counted on the top and left of the squares but not on the bottom or right side (Figure S1). A total of 99 sections were analyzed for TUNEL assay.

### Immunohistochemistry (IHC)

Cleaved caspase-3 and cytochrome c expression were detected in rat brains by IHC. IHC of cleaved caspase-3 was performed at 12 h after ethanol exposure, and IHC of cytochrome c was performed at 8 h after ethanol exposure. Briefly, after hydration, the sections were rinsed in PBS, quenched for 10 min in methanol containing 3% H_2_O_2_, and incubated for 15 min in blocking solution [PBS containing 2% goat serum, 0.2% milk, and 0.1% Triton X-100], followed by incubation overnight in primary antibodies against cleaved caspase-3 (#9661, Cell Signaling Technology, Inc., MA, USA) and cytochrome c (sc-13156, Santa Cruz Biotechnology Inc., CA, USA), diluted 1∶50 in blocking solution. After rinsing with PBS, the sections were incubated for 60 min in mouse or rabbit two-step histostaining reagent, which was used as the secondary antibody. A DAB substrate kit for peroxidase was then used to stain sections as described in the manufacturer's instructions. The secondary antibody and DAB were purchased from Zhongshan Golden Bridge Biotechnology CO., LTD., Beijing, China (PV-6001, PV-6002 and ZLI-9032). The sections were counterstained with hematoxylin.

### Scoring of cleaved caspase-3

The immunoreactive cells show different signal intensity which is dependent on the expression level of cleaved caspase-3, and the cleaved caspase-3 immunostaining signals in CPZ pretreatment groups showed a scattered and uneven distribution. To avoid the subjective error caused by selecting campus visualis, we choose the SI (staining intensity) scoring method to quantify the positive staining signals throughout the whole cortex and hippocampus.

The intensity of staining was scored as negative = SI0, weak = SI1, intermediate = SI2, or strong = SI3, correlating to absence of brown (that is, blue counterstain only), light brown, brown or dark brown staining, respectively (Figure S2). For assessment of immunohistochemical staining, the presence of any immunolabeled cells was considered to represent positive caspase-3 expression [8]. Total scores were calculated based on the SI using the following formula [9]: Total Scores = 1×n (SI1) +2×n (SI2) +3×n (SI3). A total of 81 sections were analyzed for cleaved caspase-3 immunostaining.

### Quantification of cytochrome c expression

For IHC detection of cytochrome c, the parietal cortex, CA1 of hippocampus and the thalamus were measured. The Area and IOD (integrated optical density) of cytochrome c were analyzed with Image-Pro plus 6.0, each specimen was measured randomly in 5 visual fields at a ×400 magnification, and the average mean density was calculated for each sample according to the formula: Mean Density = IOD/Area. A total of 54 sections were analyzed for cytochrome c immunostaining.

### Semi-quantitative western blot analysis

The brain sample of each rat used for western blot was taken from the whole brain. After euthanasia, tissue samples from the whole brain were rapidly dissected out and sonicated in lysis buffer (#P-0013, Beyotime Institute of Biotechnology. Shanghai, China) with phenylmethanesulfonyl fluoride (PMSF, #ST506, Beyotime Institute of Biotechnology. Shanghai, China). The samples were lysed on ice for 30 min and then centrifuged for 20 min at 12,000×g (4°C), and the supernatants were aspirated and stored at −80°C until further use. The supernatants were assayed for total protein using the bicinchoninic acid method (Enhanced BCA Protein Assay Kit, #P0010S, Beyotime Institute of Biotechnology. Shanghai, China).

Samples were resolved on 12% (actin, Bcl-2) or 15% (cleaved caspase-3, Bax, cytochrome c) Tris-glycine polyacrylamide gels using a Mini-Protean Tetra Electrophoresis System (Bio-Rad, Hercules, CA, USA). Equal amounts of total protein were boiled for 5 minutes in sodium dodecyl sulfate gel-loading buffer and applied to the gels. All gels were run with a low-range molecular weight ladder (PageRuler™ Prestained Protein Ladder, Fermentas, USA), and proteins were transferred to a nitrocellulose membrane (Millipore, Billerica, MA, USA) in a BioRad Trans-Blot SD Semi-Dry Transfer Unit. Blots were blocked with non-fat dry milk in TBS-T (10 mM Tris, 150 mM NaCl, pH 7.6, plus 0.1% Tween-20) and probed with primary antibodies as follows: rat polyclonal anti-cleaved caspase-3 antibody (#9661, 1∶1000, Cell Signaling Technology, Inc., MA, USA), which recognizes the 17 kDa cleavage product, rat polyclonal Bax antibody (sc-483, 1∶500, Santa Cruz Biotechnology Inc., CA, USA), mouse monoclonal Bcl-2 antibody (sc-7382, 1∶500, Santa Cruz Biotechnology Inc., CA, USA), mouse monoclonal cytochrome c antibody, (sc-13156, 1∶1000, Santa Cruz Biotechnology Inc., CA, USA). The immunoblots were processed with peroxidase-conjugated anti-rabbit or anti-mouse antibodies (ZB-2305, ZB-2301, 1∶2000, Zhongshan Golden Bridge Biotechnology CO., LTD., Beijing, China), and peroxidase activity was detected using the Pierce ECL Western Blotting Substrate (#32106, Thermo Fisher Scientific Inc., USA).

For densitometric analysis, immunoreactive bands were scanned with a Luminescent Imaging Analyzer (LAS-4000, Fuji, Japan) and were quantified using Multi Gauge software (Fuji, Japan). A monoclonal antibody against β-actin (sc-47778, 1∶1000, Santa Cruz Biotechnology Inc., CA, USA) was used as an internal control.

### Statistical analysis

All values are expressed as the mean ± SEM (standard error of the mean). Statistical analysis was performed using SPSS 11.0. Student's t-test was used to evaluate differences between two groups. Levene's test was used to assess the variance homogeneity (sig>0.05, homoscedasticity; sig<0.05, heteroscedasticity). One-way analysis of variance (ANOVA) was used to compare the differences between control and treatment groups, and Bonferroni's post hoc test was used for multiple comparison analysis if the variance was homoscedastic. The non-parametric Mann-Whitney U test was performed to assess the differences between treatment groups if the variance was heteroscedastic. Differences with P values<0.05 were regarded as statistically significant.

## Results

### CPZ pretreatment suppresses the apoptosis induced by ethanol in rat brains

**Figure 1:**
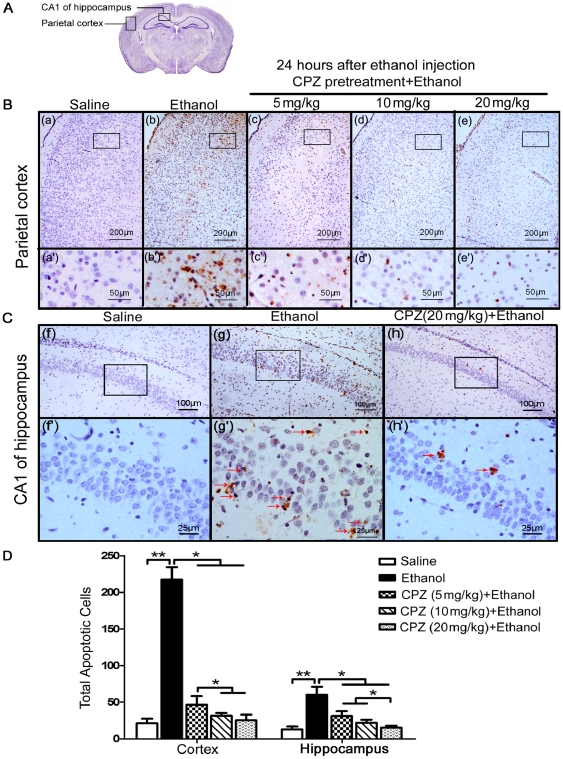
CPZ pretreatment reduces the number of apoptotic cells in rat brains after ethanol exposure. (A) The diagram shows the cross section of the brain tissue, and the regions of the parietal cortex and CA1 of hippocampus are pointed out. (B and C) TUNEL-labeled brain sections showing regions including the parietal cortex and the CA1 region of the hippocampus (stratum pyramidale). These brain sections were obtained from the following treatment groups: Saline (a, a', f and f'), Ethanol (b, b', g and g'), and CPZ pretreatment at doses of 5 mg/kg (c, c'), 10 mg/kg (d, d') and 20 mg/kg (e, e', h and h'). The apoptotic cells with brown nuclear staining could be observed in the ethanol group and CPZ pretreatment + ethanol groups. Sections were counterstained with hematoxylin. (D) The total number of apoptotic cells throughout the whole cortex and the hippocampus of each specimen were counted in three separate experiments. Values are shown as means ± SEM. A Mann-Whitney U test for multiple comparisons revealed a significant difference between the ethanol treatment group and all other groups (**P<0.01, *P<0.05, n = 6–9 animals per group).

We observed that rats treated with CPZ before ethanol administration showed a dramatic reduction of apoptosis in the parietal cortex and CA1 of hippocampus even when CPZ was administrated at a low dose (Figure 1B, C). Quantification of the number of TUNEL-positive cells throughout the whole cortex and the hippocampus showed that the apoptotic cells in ethanol group (cortex: 217.3±16.9; hippocampus: 60.0±11.1) are much more than saline group (cortex: 21.1±6.3; hippocampus: 13.0±4.1), and the administration of CPZ reduced the number of cells undergoing apoptosis caused by ethanol exposure (Figure 1D, Mann-Whitney U test, p<0.05; n = 6-9 animals per group). Data showed that in the cortex, there was a significant decrease in the number of TUNEL-positive cells in the CPZ pretreatment group at 10 mg/kg (cortex: 31.6±3.7; hippocampus: 21.8±4.3) and 20 mg/kg (cortex: 25.31±7.6; hippocampus: 15.3±2.5) doses compared with the 5 mg/kg dose (cortex: 46.5±12.0; hippocampus: 31.0±6.8) (P<0.05). In the hippocampus, CPZ (20 mg/kg) inhibited apoptosis more strongly than did the 10 mg/kg and 5 mg/kg doses (P<0.05). The results suggest that CPZ exerts a dose-dependent protective effect against the ethanol-induced apoptosis in both the whole cortex and hippocampus. Similar results were obtained in thalamus (Figure S3) showing that the ethanol-induced apoptosis (ethanol group: 154.3±20.7) could be significantly reduced by the pretreatment of CPZ at three different doses (Mann-Whitney U test, p<0.001; n = 6–9 animals per group; 5 mg/kg group: 31.3±7.4, 10 mg/kg group: 21.4±6.4, 20 mg/kg group: 21.3±5.7).

### CPZ pretreatment reduces high expression of cleaved caspase-3 induced by ethanol in rat brains

**Figure 2:**
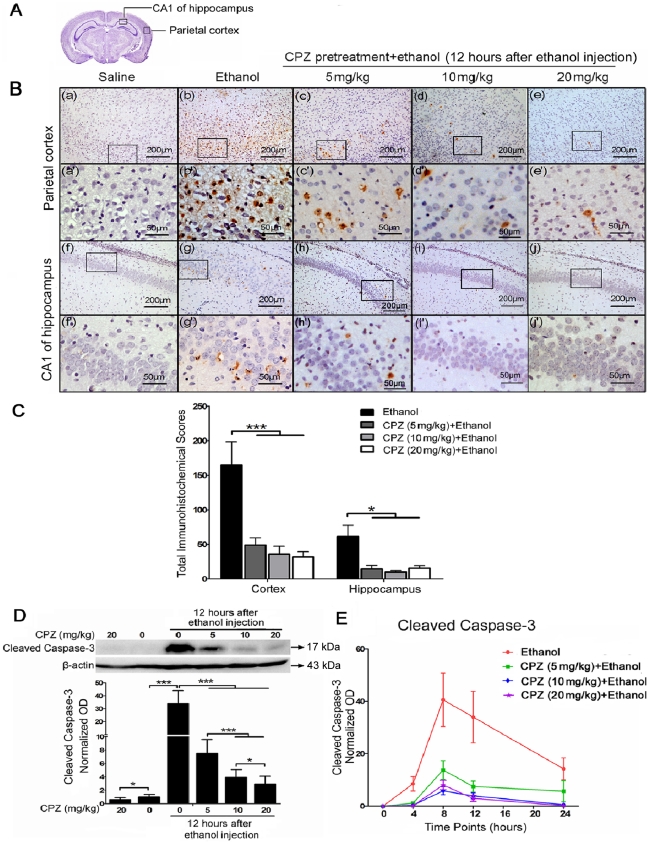
CPZ pretreatment inhibits cleaved caspase-3 expression in rat brains after ethanol exposure. (A) The diagram shows the cross section of the cerebrum, and the regions of the parietal cortex and CA1 of hippocampus are pointed out. (B) Cleaved caspase-3 labeling corresponds to brown staining could be observed in the cytoplasm and nuclei. Sections were counterstained with hematoxylin. (C) Total scores for cleaved caspase-3 immunoreactivity were calculated, and all three doses of CPZ pretreatment significantly reduce cleaved caspase-3 expression in the following regions, compared with the ethanol group: cortex (***P<0.001; n = 5–7 animals per group) and hippocampus (*P<0.05; n = 5–7 animals per group). (D) The level of cleaved caspase-3 was analyzed by western blot from the whole brain taken 12 h after ethanol administration. β-actin was used as a loading control. Cleaved caspase-3 bands were measured using optical densitometry, and the data were normalized to a control sample (defined as 1.00). Values are shown as means±SEM. Mann-Whitney U test revealed a significant difference between the ethanol group and the CPZ pretreatment groups (***P<0.001, *P<0.05, n = 4 animals per group). (E) The curves show cleaved caspase-3 levels in the ethanol and CPZ pretreatment groups at different time points (4 h, 8 h, 12 h and 24 h) after ethanol exposure. All three doses of CPZ result in a significant decrease of the cleaved caspase-3 levels compared with ethanol group.

Cleaved caspase-3 is an executioner leading to apoptosis, and often used as a marker of apoptosis [10]. Western blot and IHC were performed to determine whether inhibition of caspase-3 activation by CPZ is sufficient to prevent the apoptosis. Results showed that cleaved caspase-3 was localized in cytoplasm and nuclei with brown staining (Figure 2B). The immunoreactivity of cleaved caspase-3 is specifically expressed in the apoptotic cells; and the intensity of staining depends on the expression level of cleaved caspase-3. A significant reduction of cleaved caspase-3 immunoreactive cells was found in rats treated with CPZ before ethanol administration by scoring quantitative method. The ethanol stimuli mediated a dramatic high immunohistochemical scores in the ethanol group (cortex: 165.2 ± 33.2; hippocampus: 61.2±16.5), and a clear reduction of cleaved caspase-3 expression in both the whole cortex (Bonferroni's post hoc test, P<0.001; n = 5-7 animals per group) and hippocampus (Mann-Whitney U test, P<0.05; n = 5-7 animals per group) of the CPZ pretreatment groups (5 mg/kg: cortex 49.0±10.2, hippocampus 14.4±5.4; 10 mg/kg: cortex 36.0±11.2, hippocampus 9.8±2.1; 20 mg/kg: cortex 31.8±7.7, hippocampus 15.5±3.8) were corroborated through the quantitative analysis (Figure 2C).

Meanwhile, the expression of cleaved caspase-3 was measured by western blot using protein extracted from the whole brain of rats in each treatment group. A representative western blot depicting cleaved caspase-3 expression is shown in Figure 2D, and this blot shows proteins from groups sacrificed 12 h after ethanol exposure. The bands mainly represent the cleaved form of 17 kDa, and CPZ pretreatment significantly reduces the ethanol induced high expression of cleaved caspase-3 without any effect on the cleaved caspase-3 level at the high dose of 20 mg/kg. Densitometric quantification of the bands indicated that animals injected with ethanol alone showed a dramatic increase in cleaved caspase-3 compared with the saline control (Mann-Whitney U test, P<0.001; n = 4 animals per group). CPZ pretreatment resulted in a 5- to 10-fold dose-dependent decrease in cleaved caspase-3 expression compared with the group injected with ethanol alone (Figure 2D, Mann-Whitney U test, P<0.001); maximum inhibition was observed at 20 mg/kg CPZ. CPZ prevented ethanol-induced activation of caspase-3 at all time points tested, and at 12 h after ethanol injection, both the 10 mg/kg and the 20 mg/kg doses resulted in stronger inhibition than the 5 mg/kg dose (Figure 2D). A curve showing the dynamic effect of CPZ on activation of caspase-3 revealed that cleaved caspase-3 appeared 4 h after ethanol administration, peaked at 8 h, and was still evident after 24 h (Figure 2E). The results suggest that the ethanol induced high expression of cleaved caspase-3 could be significantly reduced by CPZ, and this finding confirmed the result obtained by TUNEL assay.

### CPZ pretreatment inhibits cytochrome c release in rat brains

**Figure 3:**
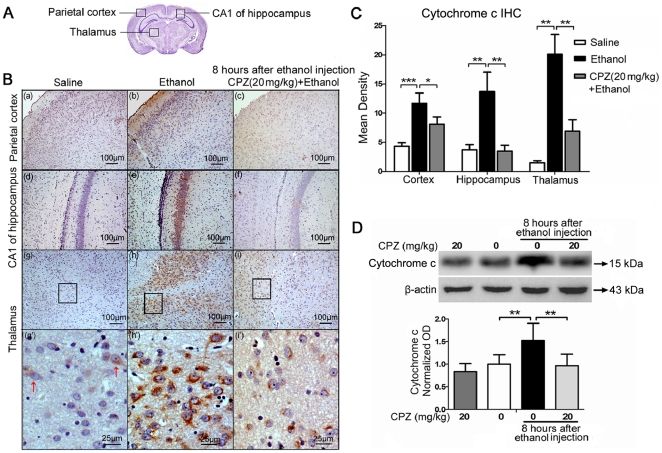
CPZ pretreatment blocks the release of cytochrome c in rat brains after ethanol exposure. (A) The diagram shows the cross section of the cerebrum, and the regions of the parietal cortex, CA1 of hippocampus and the thalamus are pointed out. (B) IHC examination of cytochrome c levels in parietal cortex, CA1 of hippocampus and thalamus of the saline control (a, d, g and g'), ethanol-treated (b, e, h and h'), and CPZ pretreatment groups (c, f, i and i'). Cytochrome c positive-immunoreactivity (brown) was located in the cytoplasm, and the inhibitory effect of CPZ on ethanol-induced cytochrome c release is clearly shown in the high magnification photos (g', h' and i'). Sections were counterstained with hematoxylin. (C) Densitometric quantification of the data yielded means ± SEM. In the parietal cortex (***P<0.001; n = 6 animals per group), CA1 of hippocampus and thalamus (**P<0.01; n = 6 animals per group), much more cytochrome c was released in the ethanol-treated group than in the saline control group. In the parietal cortex (*P<0.05; n = 6 animals per group), CA1 of hippocampus and thalamus (**P<0.01; n = 6 animals per group), the cytochrome c staining was significantly reduced in the CPZ pretreatment group compared with the group treated with ethanol. (D) Western blot analysis of cytochrome c release from the isolated cytosolic fractions of the whole brain 8 h after ethanol administration. β-actin was used as a loading control. Bands were measured using optical densitometry, and the data were normalized to a control sample (defined as 1.00). Values are shown as means ± SEM. The data indicate that CPZ pretreatment resulted in lower cytochrome c expression than that in the group injected with ethanol alone (**P<0.01; n = 4 animals per group).

Most apoptotic cell death, including ethanol-induced apoptosis, is dependent on the mitochondrial pathway, which includes regulation of Bax and release of cytochrome c from mitochondria [11]. This in turn activates caspase-3 and induces apoptosis [12], [13]. Therefore, to test whether cytochrome c is involved in the effects of CPZ, cytochrome c protein levels were detected by IHC (Figure 3B, C) and western blot (Figure 3D).

Based on previous studies, we selected the 8 h time point and the dose of 20 mg/kg as the most appropriate time point and concentration for the study of cytochrome c. Cytochrome c-positive immunoreactivity (brown) was localized in the plasm (Figure 3B). Intense staining was detected in parietal cortex, CA1 of hippocampus and thalamus of the ethanol group, and weaker staining was seen in the saline group and the CPZ pretreatment group (Figure 3B). The expression of cytochrome c could be clearly observed at thalamus as shown in high magnification images (Figure 3B). Quantification of the data (Figure 3C) showed that the mean density of cytochrome c immunostaining in ethanol group (cortex: 11.7±1.8; hippocampus: 13.7±3.4; thalamus: 20.1±3.5) is much higher than saline control (cortex: 4.3±0.6; hippocampus: 3.7±0.8; thalamus: 1.5±0.4), and the cytochrome c expression in the CPZ pretreatment group (cortex: 8.1±1.3; hippocampus: 3.5±1.0; thalamus: 6.9±2.0) was significantly lower compared to the ethanol group in regions of the parietal cortex (Bonferroni's post hoc test, P<0.05; n =  animals per group), CA1 of hippocampus and thalamus (Mann-Whitney U test, P<0.01; n = 6 animals per group).

A western blot was performed to determine the inhibitory effect of CPZ on cytochrome c release. Isolated cytosolic fractions from the whole brain were prepared according to a previously described method [14]. Quantification of the western blot indicated that a substantial release of cytochrome c occurred at 8 h after ethanol treatment (Figure 3D). Ethanol increased cytochrome c release over the saline control (Mann-Whitney U test, P<0.01; n = 4 animals per group). Importantly, CPZ pretreatment caused a significant decrease in cytochrome c release compared with ethanol alone (Mann-Whitney U test, P<0.01; n = 4 animals per group), and the level of cytochrome c declined to approximately the same amount as that in the saline control. The results of cytochrome c detection further confirmed the anti-apoptosis effect of CPZ, intimating that the action of CPZ might be exerted via the mitochondrial apoptosis pathway.

### CPZ elevates levels of the anti-apoptotic protein Bcl-2 and inhibits the increase in the Bax/Bcl-2

At the mitochondrial level, the Bcl-2 family of proteins is integral to the balance between pro-apoptotic and anti-apoptotic signaling [15], and it has been implicated in ethanol-induced apoptosis in the prenatal and neonatal developing brain [11]. In order to determine if CPZ influences the ratio between Bax and Bcl-2, which is one of the most important indices of apoptosis mediated through the intrinsic mitochondrial mechanism [16], three experiments were performed.

**Figure 4:**
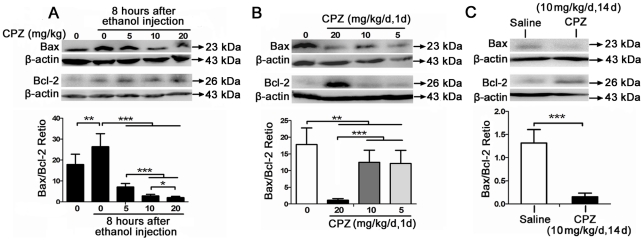
The effect of CPZ on the Bax/Bcl-2 ratio in rat brains after ethanol exposure. (A) CPZ treatment prior to ethanol exposure. The level of Bax and Bcl-2 were analyzed in the whole brain taken 8 h after ethanol administration. The Bax/Bcl-2 ratio was more strongly increased at 8 h after ethanol treatment compared to the saline control (**P<0.01; n = 4 animals per group), and pretreatment with CPZ at various doses significantly decreased the Bax/Bcl-2 ratio (***P<0.001; n = 4 animals per group). Three independent experiments were performed, and a representative blot is shown. β-actin was used as a loading control. Data are expressed as means ± SEM. (B) Single injection of CPZ. The level of Bcl-2 was examined at 24 h after a single injection of CPZ at 20 mg/kg. Groups treated with all three different doses of CPZ showed a significant decrease in the Bax/Bcl-2 ratio compared with the saline control (**P<0.01; n = 4 animals per group). In addition, the 20 mg/kg dose was the most effective at decreasing the Bax/Bcl-2 ratio (***P<0.001; n = 4 animals per group). Values are shown as means ± SEM. (C) Continuous injection of CPZ. An increase in Bcl-2 accompanied by a decrease in Bax expression was detected after continuous treatment with CPZ. Values are shown as means ± SEM. Quantification of the data showed that the Bax/Bcl-2 ratio in the group continuously injected with CPZ was significantly lower than that in the saline control (***P<0.001; n = 4 animals per group).

Firstly, rats were pretreated with CPZ (at 5 mg/kg, 10 mg/kg and 20 mg/kg) 24 h before ethanol exposure and sacrificed at 8 h after ethanol injection. Quantification of the data (Figure 4A) showed that the Bax/Bcl-2 ratio was significantly higher at 8 h after the ethanol treatment than in the saline-treated control group (Mann-Whitney U test, P<0.01; n = 4 animals per group) and that all of the doses of CPZ significantly decreased the Bax/Bcl-2 ratio compared with the group treated with ethanol only (Mann-Whitney U test, P<0.001; n = 4 animals per group). In addition, both the 10 mg/kg and 20 mg/kg doses decreased the Bax/Bcl-2 ratio more than the 5 mg/kg dose did (P<0.001; n = 4 animals per group), and the 20 mg/kg dose had the strongest suppressive effect (P<0.05; n = 4 animals per group). The data show that the effect of CPZ on the ethanol-induced increase in the Bax/Bcl-2 ratio is dose dependent.

Secondly, rats were intraperitoneally injected with a single injection of CPZ at three different doses (5 mg/kg, 10 mg/kg and 20 mg/kg) and euthanized 24 h later. Western blot analysis showed a substantial increase in the level of Bcl-2 upon treatment with CPZ at 20 mg/kg (Figure 4B). Additionally, all three CPZ doses significantly decreased the Bax/Bcl-2 ratio (Mann-Whitney U test, P<0.01; n = 4 animals per group), and the 20 mg/kg dose was more effective at decreasing the Bax/Bcl-2 ratio than the other two doses (P<0.001; n = 4 animals per group).

Finally, CPZ (10 mg/kg/d, two weeks) was intraperitoneally injected into rats continuously. The control animals were given an equal volume of saline. The results showed an increase in Bcl-2 and a decrease in Bax expression (Figure 4C) in the CPZ-treated group. The quantitated data (Figure 4C) indicated that the Bax/Bcl-2 ratio was significant lower in the group continuously treated with CPZ than in the saline control (Student's t-test, p<0.001; n = 4 animals per group).

The results for chronic and acute treatment of CPZ suggest that both the continuous use and the single use of CPZ could protect against the ethanol-induced apoptosis by leading the upregulation of anti-apoptotic Bcl-2 expression and the downregulation of Bax/Bcl-2 ratio. Significant regulatory actions on the Bax/Bcl-2 ratio could be mediated by the high dose (20 mg/kg) of CPZ acute treatment and the medium (10 mg/kg) dose of CPZ chronic treatment.

## Discussion

Over the years, the evidence regarding neuroprotection of CPZ is supported by *in vitro* and *in vivo* studies reporting its beneficial effects in various models of toxicity. It has been reported that CPZ play a dose-dependent neuroprotective effect on the ischemic spinal cord of rabbits [2]. CPZ has also been shown to reduce toxicity and Ca^2+^ uptake induced by β-amyloid protein in primary cultures of rat cortical neurons and PC12 cells [3], and protect against the cyanide-induced neurotoxicity and glutamate-induced neurotoxicity [4], [5]. In addition, a previous study showed that various atypical antipsychotics including CPZ exert a neuroprotective role against the cytotoxicity and primary hippocampal neuronal cell death induced by growth medium deprivation possibly through a caspase-dependent mechanism.[17]. However, the influence of CPZ on brain apoptosis is still unclear.

In this study, the data demonstrate that CPZ was able to protect the rat brain against the increase of apoptosis induced by ethanol. Using a TUNEL assay, we demonstrated that administration of CPZ prior to ethanol treatment can protect nerve cells against ethanol-induced apoptosis *in vivo*. The results obtained by IHC and western blot show that CPZ pretreatment leads to a lower expression of cleaved caspase-3, cytochrome c Bax, and higher expression of Bcl-2 compared to the group injected with ethanol only, suggesting that CPZ could prevent apoptosis in the brain by regulating the mitochondrial pathway. In order to evaluate the action of CPZ on the ethanol-induced apoptosis in the rat brain more comprehensive and accurate, a regional study was performed and we determined the neuroprotective effect of CPZ against ethanol induced-apoptosis in different brain regions (cortex, hippocampus and thalamus) of immature rats. The results demonstrated that the inhibitory actions of CPZ on the apoptosis in these three regions are uniform. We also performed the experiment to study the effect of CPZ on apoptosis by treating the rats with CPZ after ethanol stimuli, but no significant changes were found in the number of apoptotic cells by TUNEL assay. It might be due to the reason that the apoptotic nerve cells caused by ethanol treatment could be cleared by phagocytes within 72 h [18]. Therefore, in the current study, the rats were treated with CPZ before ethanol exposure to determine the effect of CPZ pretreatment on the occurrence of apoptosis.

It has been previously revealed that CPZ mediates an inhibitory effect on the hepatocyte apoptosis induced by the cessation of phenobarbital (Phe) treatment in mice [19], and CPZ has also been demonstrated to exert a protective role on hepatocyte apoptosis caused by D-galactosamine both in C57BL/6N Crj male mice and in cultured hepatocytes [20]. However, CPZ selectively increased cytotoxicity and induced apoptosis in leukemic cells but had no effect on the viability of normal lymphocytes or freshly isolated peripheral blood mononuclear cells [21], [22], suggesting that the influence of CPZ on apoptosis is cell type dependent.

It has been reported that the ethanol, could trigger much more apoptosis than could other pro-apoptotic agents (such as NMDA antagonists or GABA mimetics) [7]. Therefore we used seven-day-old rats injected with ethanol as a valid model of apoptosis to investigate the effect of CPZ on apoptosis in the developing rat brain. A previous study suggest that ethanol affects apoptosis in the cell differentiation and migration zones of the prenatal rat brain by modulating the mitochondrial apoptosis pathway [23].

Therefore, to clarify the mechanism involved in the protective effect of CPZ, we examined the levels of important proteins in the mitochondrial apoptosis pathway, including cleaved caspase-3, cytochrome c, Bax and Bcl-2. IHC and western blot analysis showed that CPZ blocks ethanol-induced cytochrome c release and the ensuing caspase-3 activation. Bcl-2 is a potent inhibitor of apoptosis, and cells that overexpress Bcl-2 demonstrate resistance to a variety of pro-apoptotic insults [24]. Interestingly, we found that the Bcl-2 level was increased in the brain of rats injected with CPZ either once in isolation or continuously for two weeks, which is consistent with previously published data indicating that antipsychotic treatment can raise cortical Bcl-2 levels in schizophrenic patients [6]. The Bax/Bcl-2 ratio is an important determinant of cytochrome c release from mitochondria in response to apoptotic stimuli; a high Bax/Bcl-2 ratio promotes cytochrome c release while low ratios inhibit release, and release of cytochrome c in turn activates caspase proteins. The Bax/Bcl-2 ratio appears more important than the Bax or Bcl-2 level alone in determining a cell's vulnerability to apoptosis; high Bax/Bcl-2 ratios lead to more apoptosis [25]. In this study, we found that ethanol exposure increased the Bax/Bcl-2 ratio in the developing rat brain; this result is consistent with previous findings [23], [26]. Our study demonstrated that pretreatment with CPZ attenuated the increase in the Bax/Bcl-2 ratio caused by ethanol; and that Bax/Bcl-2 ratios were significantly decreased in groups treated with CPZ in a single injection or continuously for two weeks. The chronic as well as the acute treatment of CPZ could inhibit the ethanol-induced apoptosis by decreasing the Bax/Bcl-2 ratio, and the inhibitory effect of chronic medium dose (10 mg/kg) CPZ treatment is similar to the acute high dose (20 g/kg) treatment. These data regarding the Bax/Bcl-2 ratio definitely support the results of the TUNEL assay. Based on these results, we concluded that CPZ pretreatment could protect against ethanol-induced apoptosis by regulating the mitochondrial pathway.

In addition, studies have shown that many agents exert neuroprotective effects through D2 receptors in a number of model systems, including the apoptosis of hippocampal neurons caused by transient ischemia [27], the PC12 cell apoptosis induced by H_2_O_2_ or trophic withdrawal [28], and glutamate neurotransmission and excitotoxicity [29]. In particular, CPZ, as a kind of antipsychotic drugs with high affinity for the dopamine D2 and D4 receptor subtypes, has been shown to exert a neuroprotective role against the cytotoxicity and primary hippocampal neuronal cell death induced by growth medium deprivation [17]. Furthermore, haloperidol has also been found to block the dopamine-mediated apoptosis in pituitary tumor cell lines by its antagonistic function on D2 receptors [30]. For the reason that CPZ acts primarily to block D2 receptors, we speculate that the neuroprotective effect of CPZ against ethanol-induced apoptosis might be D2 receptor dependent.

CPZ is on the World Health Organization (WHO) list of essential drugs and remains one of the drugs that are most commonly used to treat schizophrenia [31]. In addition to the dopamine hypothesis, investigators have also hypothesized that apoptosis may play a role in the pathophysiology of schizophrenia, [32]–[34]. Although it is still unknown whether or not the apoptosis is a cause or a result of schizophrenia, the previous study suggest that antipsychotic treatment can raise the anti-apoptotic factor Bcl-2 level in the schizophrenic cortexes of treated subjects compared to antipsychotic-naive subjects [6]. The leading hypothesis for the aetiology of schizophrenia remains controversial. Many research work reframed schizophrenia as a neurodevelopmental disorder [35], while other evidences in recent years suggest that the progressive course of schizophrenia is associated with ongoing neurodegenerative processes [36]. We considered that CPZ may play a role in preventing the progressive deterioration and the relapse in the course of schizophrenia by suppressing the apoptosis.

In conclusion, our study demonstrates that CPZ exerts a neuroprotective effect by decreasing mitochondrially mediated apoptosis. Although future study is necessary to determine the molecular mechanisms underlying CPZ's action and investigate other potential functions of CPZ, we propose a new consideration of CPZ as a treatment for certain chronic disorders related to apoptosis, including brain trauma, infarction and Alzheimer's disease [37]. Ultimately, if clinically relevant doses and a suitable method of administration for the prophylactic use of CPZ can be determined, it may serve as novel approach for preventing the apoptosis induced by exogenous stimuli.

## Supporting Information

Figure S1 Schematic diagram showing how the TUNEL-positive cells in the rat brain were counted. (A) All transverse sections of the brain were cut at 2 mm posterior to the bregma. (B) The slides were marked up with grid lines to separate the section into many small squares. Total numbers of the TUNEL-labeled cells were counted under the microscope with a hand-held counter. The cells touching the middle line were counted on the top and left of the squares but not on the bottom or right side.
(TIF)

Figure S2 Examples of cleaved caspase-3 scoring according to staining intensity. (A) The expression of cleaved caspase-3 was scored as negative = SI0, weak = SI1, intermediate = SI2, or strong = SI3, correlating to absence of brown (that is, blue counterstain only), light brown, brown or dark brown staining, respectively.
(TIF)

Figure S3 CPZ pretreatment inhibits the ethanol-induced apoptosis in thalamus of the rat brain. (A) The diagram shows the cross section of the brain tissue, and the region of the thalamus is pointed out. (B) TUNEL-labeled brain sections showing the region of thalamus. These brain sections were obtained from the following treatment groups: Saline (a, a'), Ethanol (b, b'), and CPZ pretreatment at doses of 5 mg/kg (c, c'), 10 mg/kg (d, d') and 20 mg/kg (e, e'). The apoptotic cells with brown nuclear staining could be observed in the ethanol group and CPZ pretreatment + ethanol groups. Sections were counterstained with hematoxylin. (C) The total number of apoptotic cells throughout the whole thalamus of each specimen was counted in three separate experiments. Values are shown as means ± SEM. A Mann-Whitney U test for multiple comparisons revealed a significant difference between the ethanol treatment group and all other groups (***P<0.001, n = 6-9 animals per group).
(TIF)

## References

[1] Carpenter WT, Koenig JI (2008). The evolution of drug development in schizophrenia: past issues and future opportunities.. Neuropsychopharmacology.

[2] Sader AA, Barbieri-Neto J, Sader SL, Mazzetto SA, Alves P (2002). The protective action of chlorpromazine on the spinal cord of rabbits submitted to ischemia and reperfusion is dose-dependent.. J Cardiovasc Surg (Torino).

[3] Ueda K, Yagami T, Asakura K, Kawasaki K (1997). Chlorpromazine reduces toxicity and Ca2+ uptake induced by amyloid beta protein (25-35) in vitro.. Brain Res.

[4] Maduh EU, Johnson JD, Ardelt BK, Borowitz JL, Isom GE (1988). Cyanide-induced neurotoxicity: mechanisms of attenuation by chlorpromazine.. Toxicol Appl Pharmacol.

[5] Stone JM, Pilowsky LS (2007). Novel targets for drugs in schizophrenia.. CNS Neurol Disord Drug Targets.

[6] Jarskog LF, Gilmore JH, Selinger ES, Lieberman JA (2000). Cortical bcl-2 protein expression and apoptotic regulation in schizophrenia.. Biol Psychiatry.

[7] Ikonomidou C, Bittigau P, Ishimaru MJ, Wozniak DF, Koch C (2000). Ethanol-induced apoptotic neurodegeneration and fetal alcohol syndrome.. Science.

[8] Konstantinidou AE, Givalos N, Gakiopoulou H, Korkolopoulou P, Kotsiakis X (2007). Caspase-3 immunohistochemical expression is a marker of apoptosis, increased grade and early recurrence in intracranial meningiomas.. Apoptosis.

[9] Schaubli M, Ritter N, Hassig M, Zerbe H, Bleul U (2008). Progesterone receptors, oestrogen receptor alpha and glucocorticoid receptors in the bovine intercaruncular uterine wall around parturition.. Anim Reprod Sci.

[10] Krajewska M, Wang HG, Krajewski S, Zapata JM, Shabaik A (1997). Immunohistochemical analysis of in vivo patterns of expression of CPP32 (Caspase-3), a cell death protease.. Cancer Res.

[11] Young C, Klocke BJ, Tenkova T, Choi J, Labruyere J (2003). Ethanol-induced neuronal apoptosis in vivo requires BAX in the developing mouse brain.. Cell Death Differ.

[12] Carloni S, Mazzoni E, Balduini W (2004). Caspase-3 and calpain activities after acute and repeated ethanol administration during the rat brain growth spurt.. J Neurochem.

[13] Chong ZZ, Lin SH, Maiese K (2004). The NAD+ precursor nicotinamide governs neuronal survival during oxidative stress through protein kinase B coupled to FOXO3a and mitochondrial membrane potential.. J Cereb Blood Flow Metab.

[14] Shichinohe H, Kuroda S, Abumiya T, Ikeda J, Kobayashi T (2004). FK506 reduces infarct volume due to permanent focal cerebral ischemia by maintaining BAD turnover and inhibiting cytochrome c release.. Brain Res.

[15] Cory S, Huang DC, Adams JM (2003). The Bcl-2 family: roles in cell survival and oncogenesis.. Oncogene.

[16] Kikuchi T, Nihei M, Nagai H, Fukushi H, Tabata K (2010). Albanol A from the root bark of Morus alba L. induces apoptotic cell death in HL60 human leukemia cell line.. Chem Pharm Bull (Tokyo).

[17] Bastianetto S, Danik M, Mennicken F, Williams S, Quirion R (2006). Prototypical antipsychotic drugs protect hippocampal neuronal cultures against cell death induced by growth medium deprivation.. BMC Neurosci.

[18] Olney JW, Tenkova T, Dikranian K, Qin YQ, Labruyere J (2002). Ethanol-induced apoptotic neurodegeneration in the developing C57BL/6 mouse brain.. Brain Res Dev Brain Res.

[19] He P, Yan ZL, Wu MC, Li LF, Guo YJ (1999). Chlorpromazine inhibits hepatocyte apoptosis caused by withdrawal of phenobarbital in mice.. Zhongguo Yao Li Xue Bao.

[20] Tsutsui S, Itagaki S, Kawamura S, Harada K, Karaki H (2003). D-galactosamine induced hepatocyte apoptosis is inhibited in vivo and in cell culture by a calcium calmodulin antagonist, chlorpromazine, and a calcium channel blocker, verapamil.. Exp Anim.

[21] Hieronymus T, Grotsch P, Blank N, Grunke M, Capraru D (2000). Chlorpromazine induces apoptosis in activated human lymphoblasts: a mechanism supporting the induction of drug-induced lupus erythematosus?. Arthritis Rheum.

[22] Zhelev Z, Ohba H, Bakalova R, Hadjimitova V, Ishikawa M (2004). Phenothiazines suppress proliferation and induce apoptosis in cultured leukemic cells without any influence on the viability of normal lymphocytes. Phenothiazines and leukemia.. Cancer Chemother Pharmacol.

[23] Lee HY, Naha N, Kim JH, Jo MJ, Min KS (2008). Age- and area-dependent distinct effects of ethanol on Bax and Bcl-2 expression in prenatal rat brain.. J Microbiol Biotechnol.

[24] Chen DF, Schneider GE, Martinou JC, Tonegawa S (1997). Bcl-2 promotes regeneration of severed axons in mammalian CNS.. Nature.

[25] Oltvai ZN, Milliman CL, Korsmeyer SJ (1993). Bcl-2 heterodimerizes in vivo with a conserved homolog, Bax, that accelerates programmed cell death.. Cell.

[26] Mooney SM, Miller MW (2001). Effects of prenatal exposure to ethanol on the expression of bcl-2, bax and caspase 3 in the developing rat cerebral cortex and thalamus.. Brain Res.

[27] Zong XM, Zeng YM, Xu T, Lu JN (2003). [Effects of D1 and D2 dopamine receptor agonists and antagonists on cerebral ischemia/reperfusion injury].. Sheng Li Xue Bao.

[28] Nair VD, Olanow CW, Sealfon SC (2003). Activation of phosphoinositide 3-kinase by D2 receptor prevents apoptosis in dopaminergic cell lines.. Biochem J.

[29] Bozzi Y, Vallone D, Borrelli E (2000). Neuroprotective role of dopamine against hippocampal cell death.. J Neurosci.

[30] An JJ, Cho SR, Jeong DW, Park KW, Ahn YS (2003). Anti-proliferative effects and cell death mediated by two isoforms of dopamine D2 receptors in pituitary tumor cells.. Mol Cell Endocrinol.

[31] Adams CE, Rathbone J, Thornley B, Clarke M, Borrill J (2005). Chlorpromazine for schizophrenia: a Cochrane systematic review of 50 years of randomised controlled trials.. BMC Med.

[32] Akbarian S, Kim JJ, Potkin SG, Hetrick WP, Bunney WE (1996). Maldistribution of interstitial neurons in prefrontal white matter of the brains of schizophrenic patients.. Arch Gen Psychiatry.

[33] Catts VS, Catts SV (2000). Apoptosis and schizophrenia: is the tumour suppressor gene, p53, a candidate susceptibility gene?. Schizophr Res.

[34] Margolis RL, Chuang DM, Post RM (1994). Programmed cell death: implications for neuropsychiatric disorders.. Biol Psychiatry.

[35] Owen MJ, O'Donovan MC, Thapar A, Craddock N (2011). Neurodevelopmental hypothesis of schizophrenia.. Br J Psychiatry.

[36] Ashe PC, Berry MD, Boulton AA (2001). Schizophrenia, a neurodegenerative disorder with neurodevelopmental antecedents.. Prog Neuropsychopharmacol Biol Psychiatry.

[37] Honig LS, Rosenberg RN (2000). Apoptosis and neurologic disease.. Am J Med.

