# Supplementary figures and images for: Chlorpromazine Protects Against Apoptosis Induced by Exogenous Stimuli in the Developing Rat Brain
Source: PLoS One. 2011 Jul 14;6(7):e21966. doi: 10.1371/journal.pone.0021966 (PMC3136481; doi:10.1371/journal.pone.0021966)

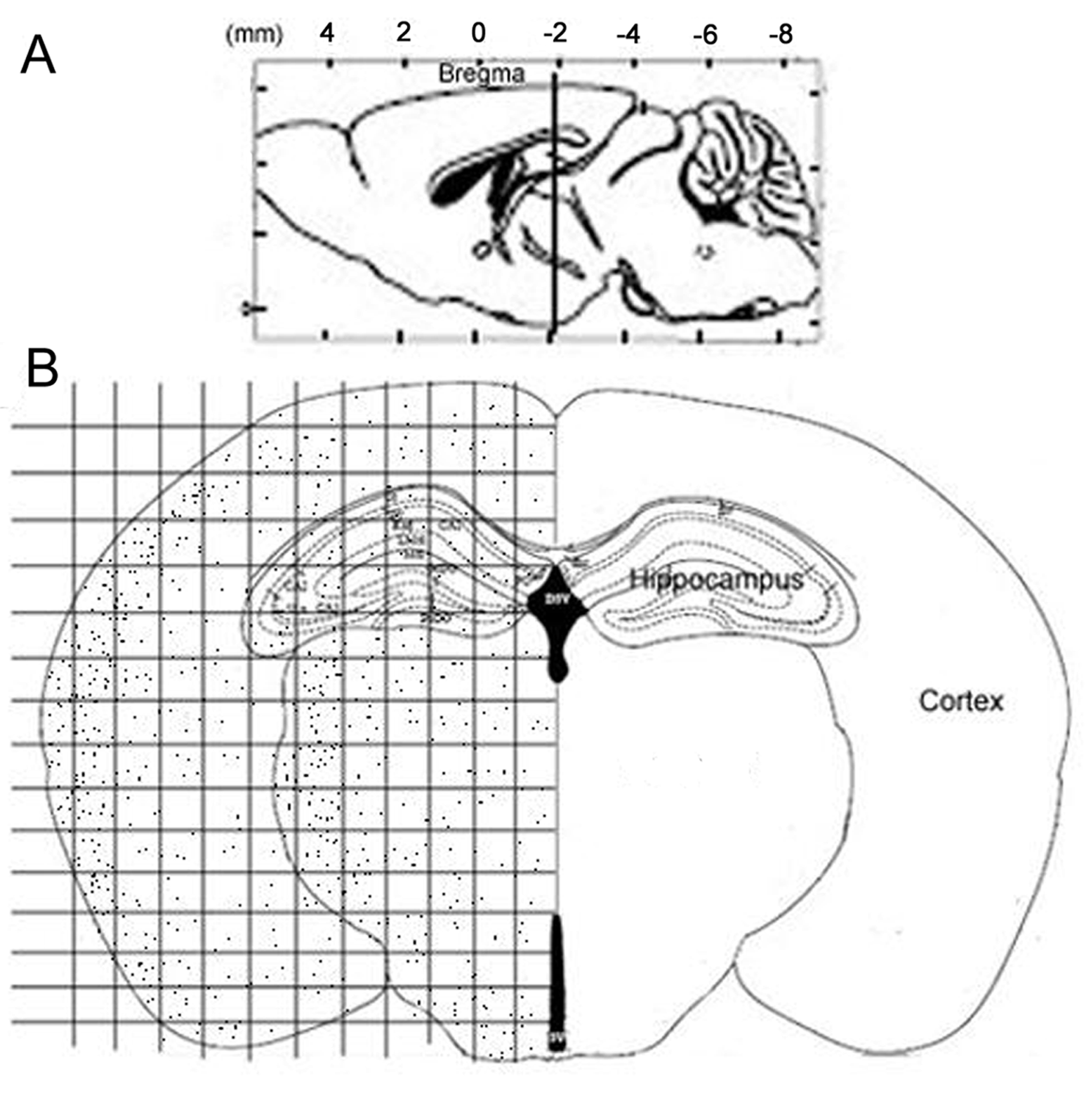

Supplement: Figure S1 — Schematic diagram showing how the TUNEL-positive cells in the rat brain were counted. (A) All transverse sections of the brain were cut at 2 mm posterior to the bregma. (B) The slides were marked up with grid lines to separate the section into many small squares. Total numbers of the TUNEL-labeled cells were counted under the microscope with a hand-held counter. The cells touching the middle line were counted on the top and left of the squares but not on the bottom or right side. (TIF) [file pone.0021966.s001.tif]

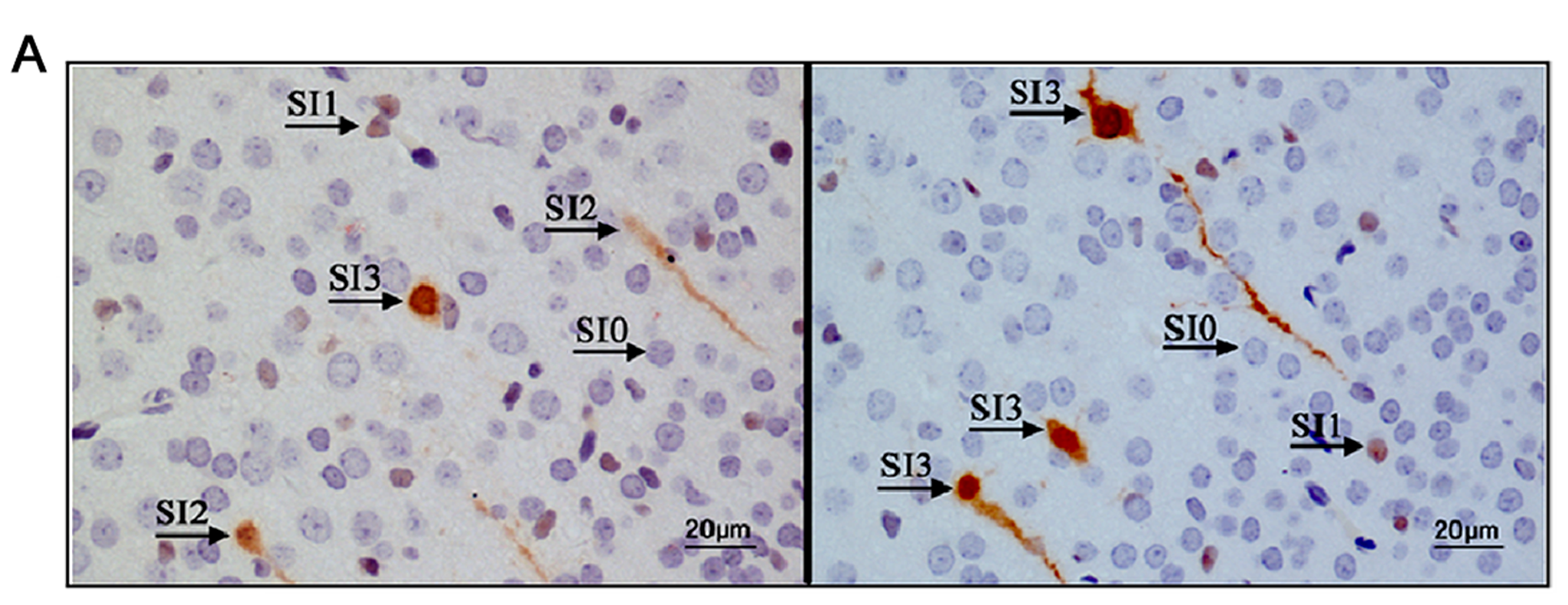

Supplement: Figure S2 — Examples of cleaved caspase-3 scoring according to staining intensity. (A) The expression of cleaved caspase-3 was scored as negative = SI0, weak = SI1, intermediate = SI2, or strong = SI3, correlating to absence of brown (that is, blue counterstain only), light brown, brown or dark brown staining, respectively. (TIF) [file pone.0021966.s002.tif]

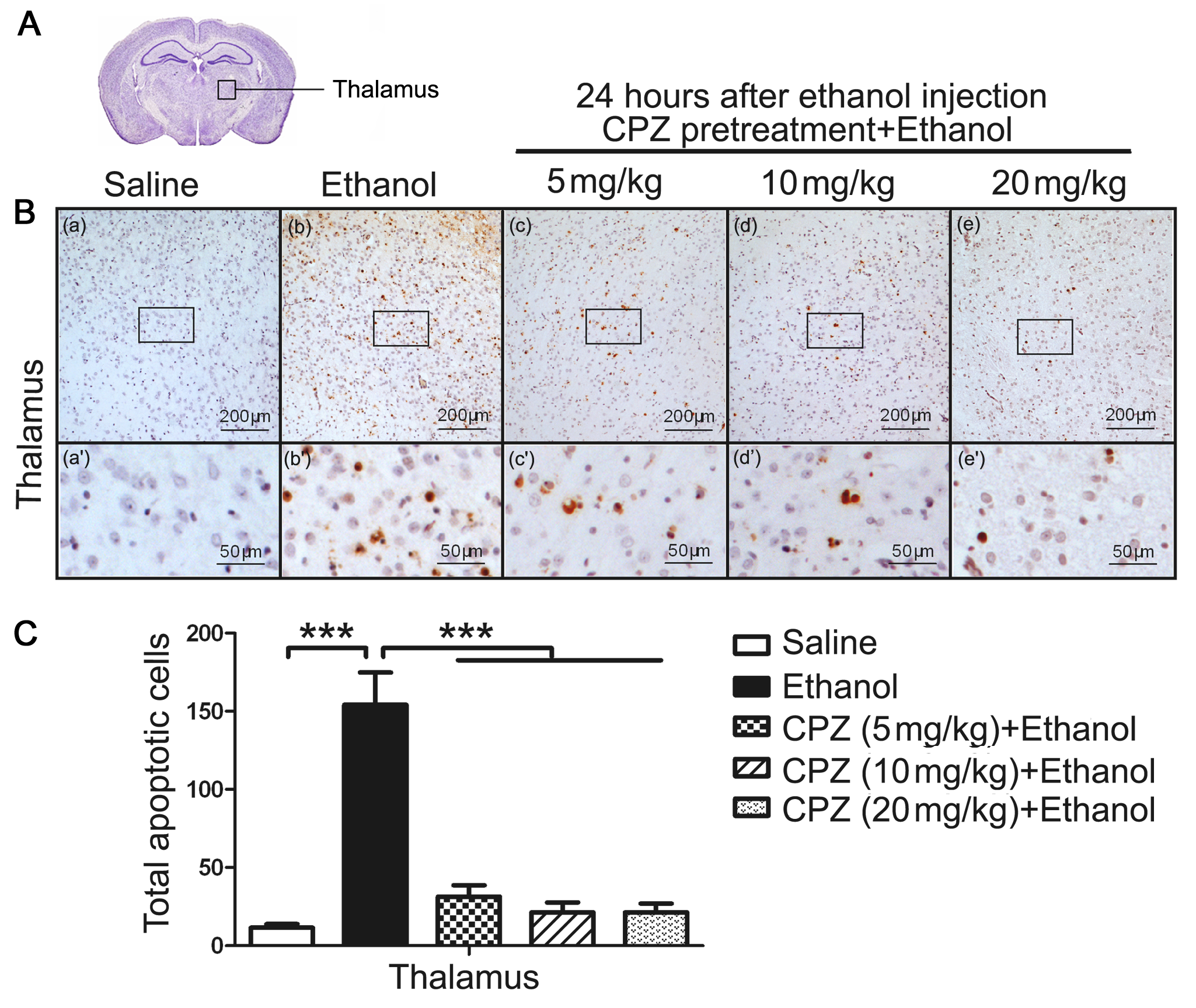

Supplement: Figure S3 — CPZ pretreatment inhibits the ethanol-induced apoptosis in thalamus of the rat brain. (A) The diagram shows the cross section of the brain tissue, and the region of the thalamus is pointed out. (B) TUNEL-labeled brain sections showing the region of thalamus. These brain sections were obtained from the following treatment groups: Saline (a, a'), Ethanol (b, b'), and CPZ pretreatment at doses of 5 mg/kg (c, c'), 10 mg/kg (d, d') and 20 mg/kg (e, e'). The apoptotic cells with brown nuclear staining could be observed in the ethanol group and CPZ pretreatment + ethanol groups. Sections were counterstained with hematoxylin. (C) The total number of apoptotic cells throughout the whole thalamus of each specimen was counted in three separate experiments. Values are shown as means ± SEM. A Mann-Whitney U test for multiple comparisons revealed a significant difference between the ethanol treatment group and all other groups (***P<0.001, n = 6-9 animals per group). (TIF) [file pone.0021966.s003.tif]
